# Consensus Integrase of a New HIV-1 Genetic Variant CRF63_02A1

**Published:** 2019

**Authors:** Y. Y. Agapkina, M. A. Pustovarova, S. P. Korolev, D. P. Zyryanova, V. V. Ivlev, A. V. Totmenin, N. M. Gashnikova, M. B. Gottikh

**Affiliations:** Lomonosov Moscow State University, Chemistry Department and Belozersky Institute of Physical Chemical Biology, Leninskie gory 1/40, 119991, Moscow, Russia; State Research Center of Virology and Biotechnology “Vector”, 630559, Koltsovo, Russia

**Keywords:** human immunodeficiency virus, CRF63_02A1 genetic variant, integrase, strand transfer inhibitor, drug resistance mutations

## Abstract

The high genetic variability of the human immunodeficiency virus (HIV-1) leads to a constant emergence of new genetic variants, including the recombinant virus CRF63_02A1, which is widespread in the Siberian Federal District of Russia. We studied HIV-1 CRF63_02A1 integrase (IN_CRF) catalyzing the incorporation of viral DNA into the genome of an infected cell. The consensus sequence was designed, recombinant integrase was obtained, and its DNA-binding and catalytic activities were characterized. The stability of the IN_CRF complex with the DNA substrate did not differ from the complex stability for subtype A and B integrases; however, the rate of complex formation was significantly higher. The rates and efficiencies of 3’-processing and strand transfer reactions catalyzed by IN_CRF were found to be higher, too. Apparently, all these distinctive features of IN_CRF may result from specific amino acid substitutions in its N-terminal domain, which plays an important role in enzyme multimerization and binding to the DNA substrate. It was also found that the drug resistance mutations Q148K/G140S and G118R/E138K significantly reduce the catalytic activity of IN_CRF and its sensitivity to the strand transfer inhibitor raltegravir. Reduction in sensitivity to raltegravir was found to be much stronger in the case of double-mutation Q148K/G140S.

## INTRODUCTION

Human immunodeficiency virus type 1 (HIV-1) has high genetic variability, resulting in the occurrence of various subtypes, circulating recombinant forms (CRFs), and unique recombinant forms (URFs) [1]. The wide variety of HIV-1 genetic variants results from the high replication rate, the tendency toward recombination and reverse transcriptase errors [2-4]. Different HIV-1 subtypes have different geographical distributions. Subtype A prevails within the former USSR [5, 6], but there are also various CRFs [7-9]. Since 2010–2012, a new HIV-1 genetic variant, CRF63_02A1, has been found to dominate in regions of the Russian Federation characterized by the highest HIV epidemic rates, such as the Kemerovo, Novosibirsk, Tomsk, and the Altai regions [10-12]. Because of its rapid spread, this genetic variant requires in-depth research.

An important step in studying a new form of HIV-1 is the characterization of its enzymes, the integrase (IN) that catalyzes the integration of viral DNA into the genome of the infected cell being one of them [13]. Three IN inhibitors – raltegravir, elvitegravir and dolutegravir – are currently used as components of antiretroviral therapy [14]. However, the emergence of viral resistance to these inhibitors has been identified [15, 16]. It is known that both mutations conferring drug resistance and the mechanisms of their occurrence in viruses of different subtypes may vary [17-22]. In this regard, it is important to study the effect of the natural polymorphism of IN on its properties.

In this study, we have characterized IN of a new HIV-1 genetic variant, CRF63_02A1 (IN_CRF), and compared it to that of HIV-1 subtype A (IN_A), which is also widespread in Russia. In particular, we have studied the influence of the structural differences between the enzymes on their DNA-binding and catalytic activities. The influence of drug resistance mutations on the catalytic and DNA-binding activity of IN_CRF, as well as its sensitivity to the strand transfer inhibitor raltegravir, has also been analyzed.

## MATERIALS AND METHODS

**Designing the consensus sequence of IN_CRF**

The HIV-1 subtype was determined using a phylogenetic and recombination analysis according to the procedure described earlier in [10, 11, 23]. The IN_CRF consensus sequence was created using the BioEdit software (Ibis Biosciences, Carlsbad, CA).

HIV-1 RNAs were isolated using a commercial Realbest DeltaMag HBV/HCV/HIV kit (Vector-Best JSC, Russia) from clinical blood plasma samples (250 μl) from two treatment-naïve patients infected with HIV- 1 variants that carried the IN genes most similar to the calculated IN_CRF consensus sequence. DNA fragments (878 bps) encoding IN_CRF were prepared by RT-PCR from the isolated RNA samples using a commercial LongRange 2Step RT-PCR kit (Qiagen, USA) and primers containing restriction sites for subsequent cloning.

**Preparation of the vector encoding IN_CRF**

DNA fragments encoding IN_CRF were ligated in the plasmid pCR_2.1Topo using the commercial TOPO® TA Cloning® Kit (pCR™2.1-TOPO®, Thermo Fisher Scientific Inc., USA). Plasmid DNA was isolated from 60 pCR_2.1Topo_IN clones (30 clones for each HIV- 1 variant) using a commercial Plasmid Purification Mini Kit (Qiagen, USA); all the DNA samples were sequenced. Plasmid pCR_2.1Topo_IN_CRF* containing an IN sequence differing from the consensus one by two amino acid substitutions was selected for further sub-cloning in expressing vector pET_15b in frame with the codons for the N-terminal His6-tag (His-tag) (Novagen, USA).

The vector pET_15b_IN_CRF with the consensus IN_CRF sequence was obtained from the vector pET- 15b_IN_CRF* by sequential site-directed mutagenesis, resulting in the amino acid substitutions I32V and I259V. The vectors encoding IN_CRF with substitutions Q148K/G140S and G118R/E138K were prepared by site-directed mutagenesis of the plasmid pET_15b_ IN_CRF using a QuikChange II Site-Directed mutagenesis kit (Agilent Technologies, USA).

The prokaryotic expression vector pET_15b carrying the gene of IN_A was a kind gift from M.G. Belikova- Isaguliants (Ivanovsky Institute of Virology, Russia).

**Preparation of recombinant proteins**

Consensus IN_CRF and IN_A proteins and those with the mutations Q148K/G140S and G118R/E138K were expressed in *Escherichia coli *strain Rosetta (DE3) (Novagen) and purified according to [24, 25]. The proteins were analyzed by 12% Laemmli PAGE, followed by staining with SimplyBlueTM SafeStain (Invitrogen, USA).

**Oligodeoxyribonucleotides**

Oligodeoxyribonucleotides U5B (5’-GTGTGGAAAATCTCTAGCAGT-3’), U5B with fluorescein residue at 5’-end (5’-Fl-U5B), U5B-2 (5’-GTGTGGAAAATCTCTAGCA-3’) and U5A (5’-ACTGCTAGAGATTTTCCACAC-3’), forming DNA substrates of IN, and all primers were purchased from DNA synthesis OJSC (Russia).

The radioactive ^32^P-label was inserted at the 5’-end of the U5B and U5B-2 oligonucleotides, and DNA substrates were formed as described in [25].

**Integrase DNA binding activity assays**

The kinetics of DNA binding by IN was studied using a fluorescence polarization assay on a Cary Eclipse Fluorescence Spectrophotometer (Varian, USA) according to [26]. The duplex 5’-Fl-U5B/U5A (10 nM) was incubated with 100 nM IN in 200 μl of buffer A (20 mm HEPES (pH 7.2), 10 mm DTT, 7.5 mm MgCl_2_) at 25°C, and the values of fluorescence polarization of fluorescein (λ_ex_ = 492 nm, λ_em_ = 520 nm) were recorded at certain time points. A curve corresponding to the time dependence of changes in fluorescence polarization was constructed, and the binding rate constant (k_on_) was calculated using the equation [IN/DNA] = [DNA]_0_ × (1-e^-kon*t^) [27].

The dissociation constant (*K*d) of the IN/DNA complex was determined using the DRaCALA method (Differential Radial Capillary Action of Ligand Assay) [28]. The U5B/U5A duplex (5 nM) with the 32P-labeled U5B-chain was incubated with IN at different concentrations (0–500 nM) in 10 μl of buffer A for 20 min at 25°C. Then, 5 μl aliquots of the mixture were applied on the AmershamTM HybondTM-ECL nitrocellulose membrane. A Typhoon FLA9500 Phosphorimager (GE Healthcare, USA) was used for membrane analysis and quantification.

**Integrase catalytic activity assays**

**Fig. 1:**
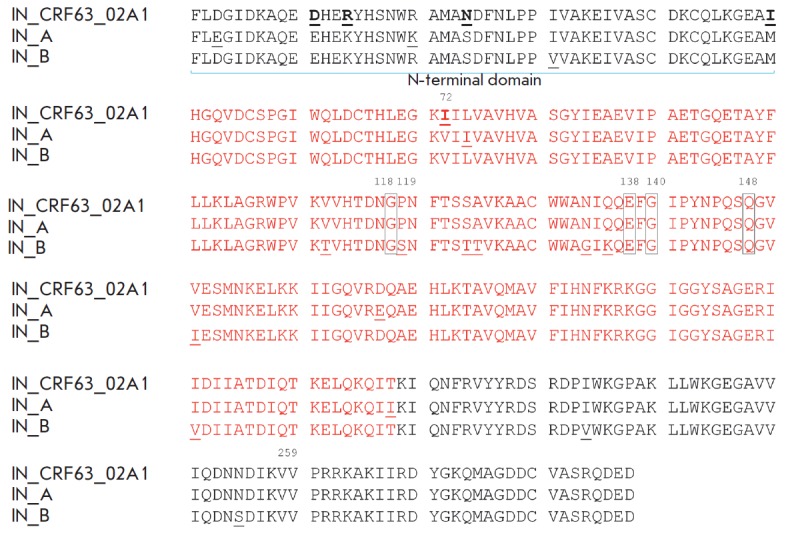
Amino acid sequences of IN_CRF, IN_A, and IN_B. The amino acids specific to IN_CRF are highlighted in bold and underlined; amino acids specific to other subtypes are underlined; amino acids whose mutations lead to drug resistance of the virus are shown with rectangles; amino acids of the catalytic domain are shown in red

For the 3’-end processing reaction, 5 nM U5B/U5A duplex (with 32P-labeled U5B-chain) was incubated with 100 nM IN in buffer A as described in [25]. The reaction products were precipitated and analyzed by electrophoresis in 20% polyacrylamide/7 M urea gel in TBE buffer. Autoradiographic data analysis was performed using a GE Typhoon FLA 9500 scanner (GE Healthcare, USA). The efficiency of 3’-processing was determined using the ImageQuantTM 5.0 software as the ratio between the intensities of the bands corresponding to the U5B substrate and the reaction product U5B-2.

When analyzing the accumulation kinetics of the 3’-processing product, the reaction mixture was incubated at 37°C. The incubation time was varied from 5 min to 7 h; the reaction efficiency versus time was plotted. The initial reaction rate was determined from the angle of inclination of the initial section of the kinetic curve (~60 min).

The dependence between the 3’-processing efficiency and substrate concentration was determined by varying the DNA concentration (0; 2.5; 4; 10; 20; 50; 100 nM). The graphs showing the reaction efficiency versus substrate concentration were plotted; the maximum reaction rate (V_max_) and the Michaelis constant (K_M_) were determined.

For the strand transfer reaction, 10 nM U5B-2/ U5A duplex (with 32P-labeled U5B-2) was incubated with 100 nM IN in buffer A for 2 and 4 h at 37°C. The reaction products were separated and analyzed as described above.

**Inhibition of the strand transfer reaction**

The strand transfer reaction was carried out as described above for 2 h in the presence of increasing inhibitor concentrations (raltegravir, Santa Cruz Biotechnology Inc., USA). The IC_50_ value was determined based on the results of three independent experiments.

## RESULTS AND DISCUSSION

**Designing the consensus IN_CRF sequence and protein purification**

IN genes from 324 HIV-1 isolates from HIV-infected treatment-naïve patients in the Siberian (*n *= 250) and Ural (*n = *74) federal districts of Russia were sequenced. Phylogenetic analysis showed that genetic variants of subtypes A (24.3%) and B (3.3%) were present, as well as recombinant forms CRF63_02A1 (55.3%) and various URFs formed as a result of secondary recombination of HIV-1 CRF63_02A1 and subtype A (6.7%).

Multiple alignment of the identified nucleotide sequences of CRF63_02A1 IN was performed, followed by translation, construction of the consensus amino acid IN sequence and its alignment with the sequences of IN from HIV-1 subtypes A and B (*Fig. 1*). Mutations typical of this HIV-1 genetic variant, e.g. E11D (93.8%), K14R (81.3%), S24N (100%), and M50I (75%), were found. The mutation L74I specific to IN_A was found only in 4% of the sequences of CRF63_02A1 IN.

**Fig. 2:**
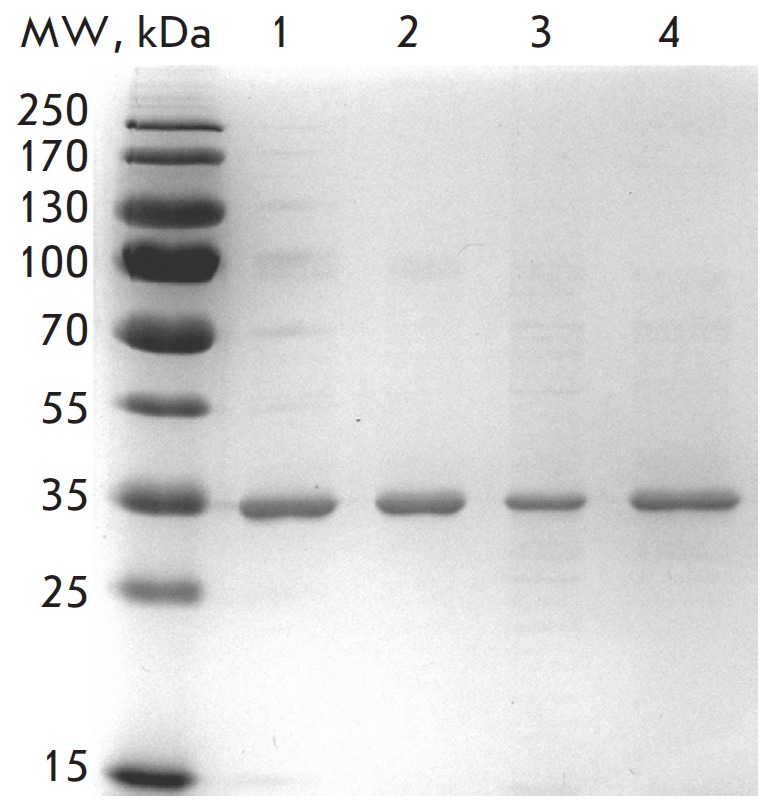
SDS-PAGE analysis of purified consensus IN_CRF and its mutant forms G118R/E138K and Q148K/ G140S. Lane *1 *– IN_CRF; lane *2 *– IN_A; lane *3 *– IN_CRF (G118R/E138K); lane *4 *– IN_CRF (Q148K/G140S); MW – molecular weight marker

Among all the studied HIV-1 IN sequences, we selected one variant that was the closest to the consensus IN_CRF. Preparation of the cDNA encoding IN_CRF and its consecutive cloning allowed us to obtain the pET-15b_IN_CRF* vector, which was then subjected to site-directed mutagenesis to introduce the I32V and I259V substitutions and to obtain the pET-15b_IN_CRF expression vector coding for a consensus IN sequence. Genetic constructions with the drug resistance mutations G118R/E138K and Q148K/G140S were obtained by site-directed mutagenesis of pET-15b_IN_CRF. The constructed vectors were used for prokaryotic expression of recombinant IN proteins and their subsequent purification on Ni- NTA-agarose to a purity ≥ 90% (*Fig. 2*).

**Characterization of DNA-binding activity**

During integration, retroviral integrases bind to the ends of viral DNA and then interact with cellular DNA; the latter interaction is not sequence-specific [29]. Recombinant HIV-1 IN protein usually has the same affinity for DNA duplexes of different structures [30]. We evaluated the capacity of IN_CRF to bind the 21-mer DNA substrate representing the terminal sequence of U5 LTR of viral DNA. The corresponding experiments with IN_A were carried out in parallel.

**Fig. 3:**
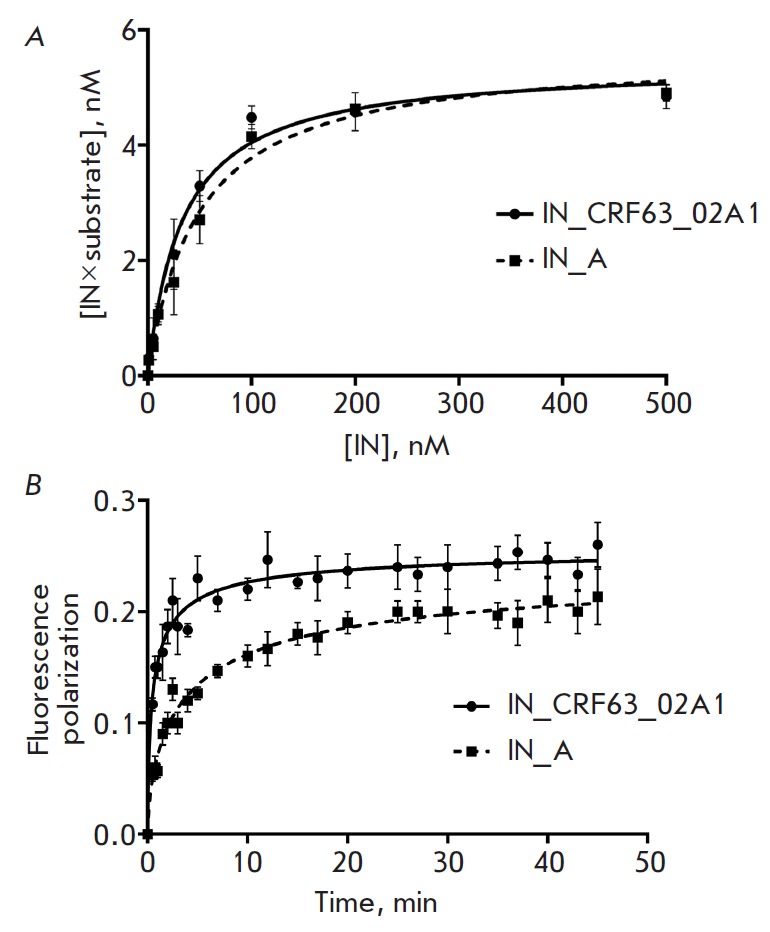
Characterization of the DNA-binding activity of IN_CRF as compared to that of IN_A. The average values of at least three independent measurements for which the standard deviation did not exceed 15% are given. *A *– The dependence between the IN/DNA-substrate complex concentration and IN concentration. *B *– The kinetics of DNA-substrate fluorescence polarization after DNA binding to IN_CRF and IN_A

Stability of the IN/DNA complex was determined using the DRaCALA method (Differential Radial Capillary Action of Ligand Assay) [28] that had been used earlier to study the complexes formed between IN_B and DNA [31]. We found that the *K*d values were similar for the complexes of DNA with both IN_CRF and IN_A (*Fig. 3A* and *Table 1*), as well as IN_B [32].

The fluorescence polarization method was applied to study the kinetics of IN binding to the fluorescein-labelled duplex 5’-Fl-U5B/U5A used as the DNA substrate. Having compared DNA binding by IN_CRF and IN_A, we found that the rate of DNA binding was higher for IN_CRF; the binding rate constants (k_on_) for these enzymes differed 2.8-fold (*Fig. 3B* and *Table 1*). The binding rate constant k_on_ for IN_A (0.24 min^-1^) was close to the value determined earlier for IN_B (0.18 min^-1^) [27].

The sequences of INs of HIV-1 subtypes A and B differ by 16 amino acid substitutions, 11 of which reside in the catalytic core; two reside in the C-terminal; and three, in the N-terminal domain. The latter three substitutions are synonymous: D3E, R20K, and V31I (*Fig. 1*). IN_CRF differs from IN_A and IN_B by four unique amino acid substitutions in the N-terminal domain: E11D, K14R, S24N, and M50I (*Fig. 1*). Keeping in mind that the rates of DNA substrate binding to IN_A and IN_B were comparable and differed significantly from that for IN_CRF, we could assume that this rate is mainly affected by the structure of the IN N-terminal domain. It is responsible for the IN multimeric state, which is crucial for its catalytic activity [33], and participates in binding of IN to the DNA substrate (viral DNA) [34, 35, 36]. There are two substitutions, S24N and M50I, in the structure of IN_CRF, which seem to be of greatest interest. The presence of an amide group in Asn and a branched chain in Ile can affect the intermolecular interactions upon formation of the catalytically active state of IN. In addition, Lys14 is in direct contact with viral DNA and plays an important role in IN multimerization [35, 37]. In IN_CRF, Lys14 is substituted with Arg. Although both these amino acids are positively charged, the Arg residue is more bulky, less hydrophobic and has a higher pKa value than Lys [38, 39]. Besides, Arg is characterized by positively charged delocalization within the guanidine group and can form multiple hydrogen bonds with different orientations [40, 41], which can contribute to DNA substrate binding.

Thus, amino acid substitutions that are inherent to IN_CRF due to natural polymorphism did not affect the stability of its complex with the DNA substrate but significantly influenced the complex formation rate.

**Table 1:** DNA-binding and catalytic activities of IN_CRF and IN_A

Characteristics	IN_CRF	IN_A
K_d_, nM	23 ± 6	25 ± 7
k_on_, min^-1*^	0.69 ± 0.09	0.24 ± 0.02
Relative efficiency of 3’-processing, %^**^	100	71
V_0_ (3’-processing), pmol/min^*^	19.3 ± 2.1	9.8 ± 2.3
V_max_, pM/min^*^	26 ± 1	16 ± 1
K_M_, nM^*^	2.6 ± 0.3	4.6 ± 0.8
V_max_/K_M_×10^3^, min^-1*^	10 ± 1	3.5 ± 0.6
Relative strand transfer efficiency, %^**^	100	77
V_0_ (strand transfer), pmol/min^*^	11.4 ± 3.2	6.5 ± 2.8

Note. The average values of at least three independent measurements (± standard deviation) are presented.

^*^P ≤ 0.05.

^**^Reaction efficiency after 300 min as compared to that for the consensus IN_CRF taken as 100%.

**Characterization of the catalytic activity of IN_CRF**

IN is involved in two successive reactions during viral replication: 3’-end processing (in which it catalyzes the cleavage of the GT dinucleotide from both 3’-ends of viral DNA) and strand transfer (insertion of the processed viral DNA into the cell DNA). Both of these reactions can be studied *in vitro *using standard protocols [25]. We used a standard synthetic DNA duplex U5B/ U5A mimicking the U5 region of HIV-1 DNA LTR for the 3’-processing reaction. The duplex contained a [5’-^32^P]-labelled U5B strand, which was turned into a product shortened by two nucleotides as a result of the reaction. In the strand transfer reaction, the [5’-^32^P]- U5B-2/U5A duplex with the already processed U5B-2 strand was used both as a substrate and a target.

**Fig. 4:**
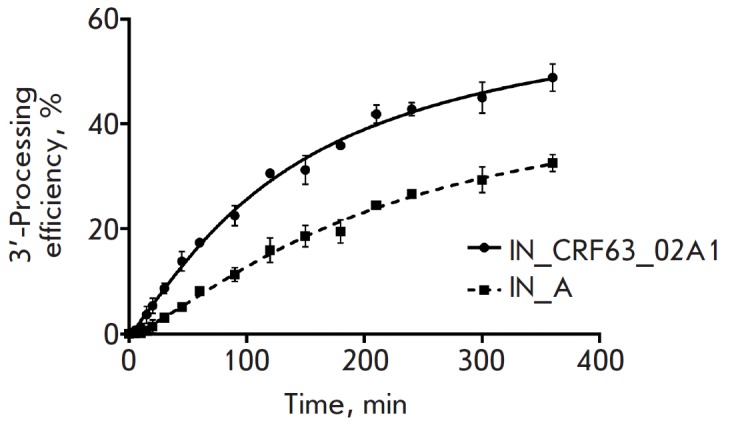
3’-Processing efficiency as a function of time

Studying the kinetics of 3’-processing showed that IN_CRF processes its substrate more efficiently and faster than IN_A does (*Fig. 4*, *Table 1*). In order to thoroughly clarify the reasons for the increased reaction efficiency, we determined the kinetic parameters of 3’-processing (*K*M and *V*_max_). It turned out that the *K*_M_ is 1.8 times lower and *V*_max_ is 1.6 times higher for IN_CRF than for IN_A (*Table 1*). Therefore, IN_CRF is characterized by a higher 3’-processing rate, which is achieved at lower substrate concentrations. Accordingly, the catalytic efficiency (*V*_max_/*K*_M_) of IN_CRF was almost three times higher than that of IN_A (*Table 1*). Obviously, such a high activity of IN_CRF cannot be attributed only to the higher rate of the DNA substrate binding (*Fig. 3*), especially taking into account that the dissociation constants for the complexes of both enzymes with DNA were similar. As mentioned above, the N-terminal domain of IN is responsible for its multimeric state, which changes when IN binds to its DNA substrate [42, 43] to form the catalytically active enzyme-substrate complex. It is possible that amino acid substitutions located on the N-terminal domain of IN_CRF contribute to the formation of such a complex, thereby stimulating the more efficient reaction.

**Fig. 5:**
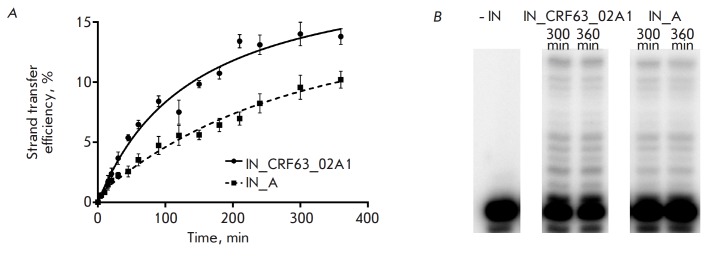
Characteristics of the strand transfer reaction catalyzed by IN_CRF and IN_A. The average values of at least three independent measurements for which the standard deviation did not exceed 15% are given. *A *– Strand transfer kinetics. *B *– The products of the strand transfer reaction for IN_CRF and IN_A (electrophoretic analysis of the reaction products after 300 and 360 min)

When studying the strand transfer reaction, we determined the reaction efficiency and rate, as well as the pattern of the reaction products, which demonstrates on which site the substrate is inserted into the target DNA. Strand transfer efficiency and rate were again higher for IN_CRF, whereas the pattern of products was the same (*Fig. 5*). Of note, the profiles of the strand transfer products were different for IN_A and IN_B [25]. The profiles of the integration products can vary if the modes of complex formation between IN and target DNA differ. The catalytic and especially C-terminal domains of IN are known to be mainly involved in the target DNA binding [36]; their structures are similar for IN-CRF and IN_A and significantly differ from that of IN_B (*Fig. 1*). Therefore, location of the target DNA in its complexes with IN_CRF and IN_A is similar and differs from its location in the complex with IN_B.

**The influence of drug resistance mutations on IN_ CRF activity and its sensitivity to raltegravir**

Since no data are available about drug resistance mutations in the genetic variant of the virus under study, we introduced mutations known to confer resistance to strand transfer inhibitors in other HIV-1 subtypes in the IN_CRF gene. We chose the primary mutation Q148K and the secondary compensatory mutation G140S causing IN resistance to raltegravir and elvitegravir [44, 45]. The G118R and E138K substitutions resulting in reduced IN sensitivity to dolutegravir were also selected [46, 47]. Therefore, IN_CRF protein variants containing Q148K/G140S and G118R/E138K double substitutions were prepared (*Fig. 2*). We investigated their DNA binding activity and the dependence of their 3’-end processing efficiency on the IN concentration and reaction time. It turned out that the introduced mutations did not affect the stability of the enzyme-substrate complex but significantly reduced the IN catalytic activity (*Table 2*). Interestingly, the Q148K/G140S double substitution reduced the IN_ CRF activity more significantly than G118R/E138K did. The initial rate of 3’-processing for the mutant IN proteins decreased 7.1- and 3.4-fold, respectively, as compared to that for the initial consensus IN_CRF. We had previously revealed a decline in the catalytic activity of IN_A in the 3’-processing reaction resulting from the Q148K/G140S and G118R/E138K mutations, but this decline was the same for both double mutations (3.8-fold) [25].

**Table 2:** DNA-binding and catalytic activities, as well as raltegravir resistance of IN_CRF, IN_A, and their mutant forms Q148K/G140S, G118R/E138K

Characteristics	IN_CRF			IN_A		
	consensus	Q148K/ G140S	G118R/ E138K	consensus	Q148K/ G140S^*^	G118R/ E138K^*^
K_d_, nM	23 ± 6	28 ± 9	25 ± 5	25 ± 7	ND	ND
V_0_ (3’-processing), pmol/min	19.3 ± 2.1	2.7 ± 0.4	5.6 ± 1.2	9.8 ± 2.3	2.6 ± 0.1	2.6 ± 0.4
Relative efficiency of 3’-processing, %	100	22	31	100	25	24
Relative strand transfer efficiency, %	100	27	22	100	20	23
Raltegravir IC_50_, nM	7 ± 2	500 ± 50	50 ± 3	5 ± 2	400 ± 150	7 ± 3
FC	1	71	7	1	80	1.4

^#^The structure was solved by NMR in contrast to the other structures solved by X-ray crystallography.

**Fig. 6:**
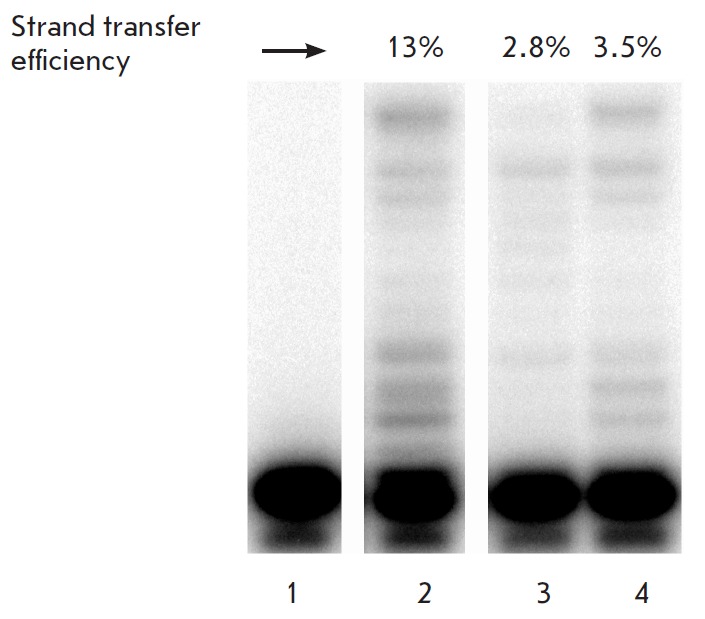
Electrophoretic analysis of the strand transfer reaction products for IN_CRF (lane *2*) and its mutants G118R/ E138K (lane *3*) and Q148K/G140S (lane *4*) (reaction time 300 min). Lane *1 *– DNA control without IN added. The reaction efficiency is shown above the gel

Similarly to IN_A [25], the Q148K/G140S and G118R/E138K substitutions significantly reduced the efficiency of strand transfer catalyzed by IN_CRF (*Table 2*). The decrease was slightly stronger in the case of G118R/E138K than for the Q148K/G140S mutation: 4.6-fold versus 3.7-fold, respectively. Interestingly, G118R/E138K substitutions in the case of IN_B affected the strand transfer efficiency very slightly [25]. The strong negative effect of these substitutions in both IN_CRF and IN_A is obviously related to the natural polymorphism S119P (*Fig. 1*), resulting in the more rigid conformation of the active center and the reduced ability of both integrases to adapt to the G118R mutation. We assume that it is the rigid conformation of the active center within IN_CRF and IN_A bearing the G118R/E138K double mutation that limits their ability to bind to the target DNA, thus resulting in a sharp decrease in the number of strand transfer products for the G118R/E138K mutants (*Fig. 6* and [25]). The Q148K/G140S substitutions did not change the pattern of the reaction products when compared to the initial IN_CRF (*Fig. 6*).

We also studied the sensitivity of IN_CRF and its Q148K/G140S and G118R/E138K mutants to inhibition by raltegravir, the drug used for treatment of HIV-infected patients in the Russian Federation. IN_CRF was efficiently inhibited by raltegravir (*Table 2*); the IC_50_ value was close to those obtained earlier for IN_A and IN_B [25]. Introduction of Q148K/G140S resistance mutations detected in other HIV-1 subtypes also led to the emergence of IN_CRF resistance. We observed a 70-fold increase in the IC_50_ value, which was consistent with the data previously obtained for IN_A [25]. The G118R/E138K mutations also reduced the sensitivity of IN_CRF to raltegravir but not substantially (FC = 7, *Table 2*). It should be noted that IN_A bearing G118R/E138K mutations exhibited almost no drop in sensitivity to raltegravir [25].

## CONCLUSIONS

The recombinant IN protein from a new HIV-1 genetic variant, CRF63_02A1, that is rapidly spreading across Siberia has been identified and characterized for the first time. IN_CRF was found to catalyze both 3’-processing and strand transfer reactions faster and more efficiently than IN of HIV-1 subtype A does. The high rates of these reactions are likely to be ensured by faster binding of the substrate DNA and the higher catalytic efficiency that were found for IN_CRF. Apparently, all these changes could be attributed to the E11D, K14R, S24N, and M50I amino acid substitutions residing in the N-terminal domain of IN_CRF, which plays an important role in IN multimerization and binding to viral DNA. However, due to the lack of significant differences in the catalytic and C-terminal domains of IN_CRF and IN_A, the pattern of the strand transfer products characterizing a mode of the target DNA positioning in the active site of the enzyme-substrate complex was similar for both INs.

The results obtained allowed us to suggest that the resistance of the HIV-1 genetic variant CRF63_02A1 to raltegravir may develop primarily due to the emergence and fixation of Q148K/G140S mutations as it was described for other HIV-1 subtypes [48]. The introduction of these mutations in IN_CRF led to a 70-fold increase in resistance to this inhibitor as compared to the initial parental IN_CRF. The G118R/E138K mutations resulted in only a seven-fold increase of the resistance of IN_CRF to raltegravir.

## Abbreviations

HIV-1: human immunodeficiency virus type 1
IN: integrase
IN_CRF: integrase of the HIV-1 genetic variant CRF63_02A1
IN_A: HIV-1 subtype A strain FSU-A integrase
IN_B: HIV-1 subtype B strain HXB-2 integrase
CRF: circulating recombinant forms of HIV-1
URF: unique recombinant forms of HIV-1
IC50: inhibitor concentration suppressing enzyme activity by 50%
FC: change in the IC50 value for mutant proteins compared to the wild type
RT-PCR: reverse transcription polymerase chain reaction
SDS: sodium dodecyl sulfate
PAGE: polyacrylamide gel electrophoresis
DTT: dithiothreitol
